# Socio-demographic characteristics and the utilization of HIV testing and counselling services among the key populations at the Bhutanese Refugees Camps in Eastern Nepal

**DOI:** 10.1186/s13104-018-3657-2

**Published:** 2018-07-31

**Authors:** Salina Khatoon, Shyam Sundar Budhathoki, Kiran Bam, Rajshree Thapa, Lokesh P. Bhatt, Bidhya Basnet, Nilambar Jha

**Affiliations:** 1 United Nations High Commissioner for Refugees, Sub-Office, Damak, Nepal; 2 School of Public Health and Community Medicine, B P Koirala Institute of Health Sciences, Dharan, Nepal; 3 Local Action for Global Health and Environment (LAGHE)-Nepal, Dhangadhi 5, Kailali, Nepal; 4 AMDA Nepal Primary Health Care Project for Bhutanese Refugees, Damak, Nepal; 5 Association of Medical Doctors of Asia-HIV/AIDS for Migrants (AMDA-HAMI), Kathmandu, Nepal; 6 Birat Health College and Research Center, Biratnagar, Nepal

**Keywords:** HIV testing and counselling, Refugees in Nepal, Utilization of HIV testing and counselling services, Bhutanese refugees

## Abstract

**Objectives:**

This cross-sectional study was conducted to describe the socio-demographic characteristics, assess the utilization of HIV testing and counselling services, and to explore the reasons for the non-utilization of HIV testing and counselling services among the key populations at the Bhutanese refugee camps in eastern Nepal.

**Results:**

The HIV testing and counselling services are utilized by less than a third (29%) of the key population among the Bhutanese Refugees. The prime source of information about the HIV testing and counselling sites has been health workers followed by peer/outreach educators and neighbors. Common self-reported barriers for utilization of HIV testing and counselling services by the Bhutanese refugees were self-perceived stigma about HIV, the fear of being discriminated and the lack of knowledge about HIV testing and counselling services. There is a need to analyze the gap between availability and utilization through more qualitative approaches in order to identify interventions to increase the uptake of the HIV testing and counselling services.

## Introduction

HIV remains a major public health problem worldwide. Almost 78 million people have been infected and about 39 million have died because of HIV infection since the beginning of the epidemic. As of 2016, there were an estimated 36.7 million (34.0 million–39.8 million) people living with HIV (PLHIV) globally, including almost 1% of adults aged 15–49 years [1]. While HIV epidemiological trends have begun to reverse in the Southeast Asian region, the HIV prevalence has remained constant at < 0.1% [2].

Refugees are at risk of contracting HIV due to the extended displacement and associated disruption to their lives. Although HIV services exist, several challenges limit access of refugees to services [3]. Refugees are often accused of importing HIV to the countries of asylum, thus discriminated [4]. Refugee women are forced to engage in commercial sex for food, shelter material and other basic commodities [5], whilst refugee men who leave their partners behind often engage with commercial sex workers, both inside or outside refugee camps, and are therefore placed at risk of HIV infection [6, 7].

HIV testing and counselling (HTC) is an evidence-based, cost-effective intervention to prevent HIV infection [8]. HTC motivates people to modify behaviors in order to prevent HIV infection or transmission [9, 10]. HIV/AIDS is also a priority sector in the new Sustainable Development Goal 3 [11] and HTC services have been widely promoted in low-middle income countries, as part of their primary health care [12, 13], and can help government in HIV policy making [14]. The success of the intervention hinges upon access and utilization of the HTC services [8, 15, 16].

The relationships between population migration and situations that can increase the risk of HIV infection are well documented [17]. HIV testing is lesser a priority compared to the needs of meeting the daily survival needs among refugees in Uganda [18]. As the HTC services are made available through a HIV section in the health center of each Bhutanese refugee camp [19], it is also very crucial to make sure they are utilized [18].

HIV positivity among the Bhutanese refugees varied from 0.23% in 2009 and 0.03% in 2010 to 0.12% in 2011 [20]. Health services along with HTC services are delivered through a permanent clinic based unit in the primary health center of the Bhutanese refugee camp in Nepal [21, 22]. Understanding the individual, local and socio-cultural aspect of HTC services utilization is useful for the programs in similar settings. It is imperative to explore the utilization of HTC among such vulnerable population [23]. Utilization of HTC services by the key population of the Bhutanese refugee camps is an important step in prevention and control HIV among these populations. As the Bhutanese refugees have been migrating to many countries around the world [17], it is crucial to have a strong HIV control program with high uptake of HTC services in Nepal.

With limited availability of HTC utilization literatures regarding the refugee and displaced population from around the world and no literature found from Nepal, this study could provide basic information on the HTC utilization among the Bhutanese refugees in Nepal. The evidence generated from this study will be useful for HIV programs in refugee settings Nepal and also around the world. This study thus aims to describe the socio-demographic characteristics, assess the utilization of HTC services, and explore the reasons for the non-utilization of HTC services among the key populations at the Bhutanese refugee camps in eastern Nepal.

## Main text

### Methods

We conducted a cross sectional study using quantitative methods in Beldangi and Sanischare refugee camps in eastern Nepal from August to December 2015.

As of August 2015, the total number of refugees from Bhutan living in Beldangi and Sanischare camps was 20,051 [21]. Using a snowball sampling technique [24], 323 respondents were identified as key populations at risk of HIV infection. All 323 key population identified in the study were approached for the study and all of them agreed to participate, giving a response rate of 100% in this study. A semi structured questionnaire was prepared adapting the tool from the previous study [3].

Having ever accessed HTC services and completed a session was considered a positive response for utilization. The interviews were carried out in Nepali by the research team. Bivariate analysis was done using Chi square test for the associated factors, and Odds ratio was calculated along with its 95% confidence interval. Multivariate analysis was done further for the associated factors with p-value < 0.25 in bivariate analysis, using binomial logistic regression analysis, for adjusted odd’s ratio and the confidence interval. The p-value of < 0.05 was considered as statistically significant.

### Results

The key population included, the primary target population including migrant workers (36.5%), intravenous drug user (11.1%), client of sex worker (10.5%), female sex workers (7.1%) and men having sex with men (2.8%); and the secondary target population including spouse of primary target population (31.9%).

Almost all (99%) lived within 30 min walk of the HTC center. The study found the utilization of HTC services was 29%. The bivariate analysis showed significant association of utilization of HTC services with the age and literacy status (Table 1). Sex, religion, marital status and employment status were not found to be associated significantly.

**Table 1:** Respondent characteristics associated with the utilization of HTC services

Socio-demographic characteristics	Utilization of HTC services		Unadjusted odds ratio			
	Yes (%)	No (%)	OR	95% CI (lower)	95% CI (upper)	p-value*
Age (years)						
< 25	54 (25.4)	159 (74.6)	1	1.06	2.86	*0.026*
≥ 25	41 (37.3)	69 (62.7)	1.74			
Sex						
Female	36 (25.7)	104 (74.3)	1	0.84	2.24	0.248
Male	59 (32.2)	124 (67.8)	1.37			
Religion						
Hindu	33 (33.3)	66 (66.7)	1			0.453
Buddhist	27 (28.7)	67 (71.3)	0.80	0.43	1.48	
Christian	11 (24.4)	34 (75.6)	0.64	0.29	1.43	
Kirat	20 (25.6)	58 (74.4)	0.68	0.35	1.33	
No religion	4 (57.1)	3 (42.9)	2.66	0.56	12.61	
Marital status						
Married	42 (27.3)	112 (72.7)	1	0.75	1.97	0.495
Single	53 (31.4)	116 (68.6)	1.21			
Literacy status						
Literate	75 (25.9)	215 (74.1)	1	2.01	9.03	*<* *0.001*
Illiterate	20 (60.6)	13 (39.4)	4.41			
Employment status						
Unemployed	51 (26.3)	143 (73.7)	1	0.89	2.35	0.166
Employed	44 (34.1)	85 (65.9)	1.45			
Type of key population						
Primary	66 (30.0)	154 (70.0)	1	0.54	1.53	0.734
Secondary	29 (28.2)	74 (71.8)	0.91			

* Calculated using χ^2^ test

All the variables which were found to be associated with 25% significance (p ≤ 0.25) in bivariate analysis were taken for multivariate analysis. Age, sex, literacy and employment status were considered for multivariate analysis (Table 2). Literacy status was found to be significantly associated with HTC utilization even after adjusting for other confounders (p < 0.001).

**Table 2:** Multivariate analysis of characteristics associated with utilization of HTC services

Characteristics	Category	Beta-coefficient	p-value	Adjusted odds	95% CI for adjusted odds	
					Lower	Upper
Age (years)	< 25	1				
	≥ 25	0.225	0.416	1.252	0.728	2.153
Sex	Female	1				
	Male	0.275	0.296	1.316	0.786	2.205
Literacy status	Literate	1				
	Illiterate	1.462	*0.000*	4.316	1.951	9.549
Employment status	Unemployed	1				
	Employed	0.375	0.160	1.455	0.863	2.455
Constant		− 1.449	0.000	0.235		

Among 323 respondents, 228 (70%) knew about the HTC services. The sources of information about the HTC services among them were health workers at the camps (51.7%), peer/outreach educators (44.6%), neighbors, mass media including radio/television (16.4%), family/friends (14.6%) and community education sessions (14.6%).

Self-perceived stigma about HIV was the most frequently reported reason (55%) for not visiting the HTC center. Respondents believed that visiting the center would foster and communicate negative images about them in the community. Other reasons given for not visiting the HTC center are listed in Table 3.

**Table 3:** Reasons for not visiting the HTC center (n = 228)

Reasons^a^	% of respondents
Self-perceived stigma about HIV	55
Fear of discrimination	54
Lack of knowledge about HTC services	47
Perception of being at low or no risk	43
Fear of rejection by spouse/family members	34
Fear of HIV positive test result	30
Fear of violence if tested HIV positive	23
Fear of community people finding out	17
Not believing that testing will help	10
Fear of delay in resettlement process if found positive	7
Fear of needles	6
Not accepting that HIV as a disease exists	6
Not having the patience to wait for the results	5
Inconvenient testing hours	4
The perception that the test is expensive	3
Perceived long waiting time	3
Inconvenient location of HTC center	2

^a^The responses were not mutually exclusive

### Discussion

HTC services allow people to assess their risk behaviors, identify their HIV status and discuss HIV related issues with skilled counsellors. Refugees are particularly vulnerable to HIV infection as conflicts, insecurity, and poverty offer a fertile ground for HIV transmission [25]. Utilization of existing HTC services by the refugees provides benefits for the refugee community, as well as the host population in Nepal and the resettled population around the world.

The HTC services were utilized by only a little over a quarter of the key populations (29%) identified in this study. The utilization of HTC services varied among different population groups around the world. Utilization of HTC services was 94% among health workers in Zimbabwe [26], 32–63% among university students in Kenya [27, 28], and 87% among men who have sex with men (MSM) in Nepal [29]. However, utilization data among refugees from elsewhere were not found. Interventions to increase the utilization of HTC services among key populations among the Bhutanese refugees may need further exploration. People who are younger than 25 years use the HTC center at the camps less than those who are 25 years and older. This shows a need for target programs to meet the needs of young refugees. Education was the only socio-demographic characteristic that was significant in the multivariate analysis. It was notable that illiterate rather than literate refugees used HTC services within the camps more frequently. The findings corroborates with the findings from findings from key populations in South Africa [30], where the illiterate people were more likely to utilize the HTC services. The underlying reasons for this phenomenon may need further exploration, perhaps using qualitative methods.

Health workers and outreach educators were the major source of information about the HTC services. A third of the respondents were not aware of the services, highlights the needs for further exploration to expand awareness programs. A fifth of respondents learnt about HTC through mass media such as television and radio. However, in Ghana, 2/3rd of respondents learnt about similar services through these channels [31]. While in Nepal there is a frequent radio show embedded in the HIV program for Bhutanese refugees, its role delivering HTC-related information seems modest [32].

Stigma, discrimination and the issues reported as barriers demand clear addressing [14, 23]. Identifying the socio-cultural dimensions of HIV as a disease and its treatment as perceived by the Bhutanese refugees remains to be explored. The stigma surrounding HIV and the people living with HIV serves as an additional barrier, affecting the acceptability of testing [33, 34] and the extent of social support [35]. Many people fear the psychosocial consequences of testing positive for HIV, particularly when it may lead to loss of social status, discrimination [36–38], domestic violence or even abandonment [39]. Fear of being HIV-positive and the perception of being at low risk are areas that are linked with awareness and the knowledge about HIV and its transmission. These are also the reasons for self-stigma. The findings from this study resonate with previous research [31, 40]. A study from 2016 reports family planning service provided by health centers to the Bhutanese refugees to have a good uptake along with the refuges being well informed about the family planning services [22]. The enabling factors from the family planning services could be further explored to find ways to promote the HTC services among the refugee population.

Barriers to utilization of HTC services may be related to lack of knowledge and awareness of issues related to the prevention and management of HIV. Newer ways to educate the people may be needed. The concern of confidentiality of the information, i.e. fear of rejection if spouse finds out and the fear of discrimination is community people find out reported in this study matches with the findings from another study [14] in Nepal. Confidentiality concern is a perceived barrier for the uptake of HTC services in other settings as well [41]. HTC staff is important to build the client confidence for HIV testing and counselling and they could play a greater role in minimizing the stigma. The quality of counselling by the counsellors as well as adherence to the principles of confidentiality may need to be further explored [23]. Long waiting time and fear of people finding out about their status were perceived reasons for not utilizing the HTC services in this study [40, 42]. The health service barriers could have strong influence on the utilization of HTC services as reported in other studies [40]. There could be possibilities of incorporating mobile clinic based HTC services [43], however, further research and consultation may be required.

The perceived fear that their resettlement process will be interrupted if tested positive, may be an important reason for Bhutanese refugees. The third country resettlement for Bhutanese refugees is ongoing since 2007 and the HIV testing was a routine before departure. However, in the recent years, the testing is voluntary and refugees are not stopped on the grounds of being HIV positive [44]. The policy on HIV testing for resettlement also may discourages compulsory testing and mandates the use of standard HTC services for any refugees opting for resettlement [45]. Apparently, there seems a communication gap about this information to the refugee community.

### Conclusion

The utilization of HTC services was low. Culturally appropriate interventions may be needed to address the perceived stigma and discrimination of HIV among refugees. Comprehensive communication strategies may be developed to disseminate information about HIV testing optimally with the outreach and peer educators as a key source of information and facilitators of communication.

## Limitations

Snowball sampling may have introduced community bias. The potential key population member that are not connected to local peers may have been missed out as the sample recruitment in snowball sampling is strongly respondent driven. The cross-sectional nature of the study also fails to provide the casual relationship between factors and the utilization of HTC services. The use of open questions has pointed out the reasons for not using the services; however, a robust qualitative design in the future may provide in-depth understanding.

## Abbreviations

HTC: HIV testing and counselling
PLHIV: people living with HIV
